# Challenging Dogma about Myonuclei Behavior in Skeletal Muscle Cells

**DOI:** 10.1093/function/zqac068

**Published:** 2022-12-26

**Authors:** Espen E Spangenburg

**Affiliations:** Department of Physiology, Brody School of Medicine, East Carolina University, 600 Moye Blvd Greenville, NC 27834, USA; East Carolina Diabetes and Obesity Institute, East Carolina University, 115 Heart Dr, Greenville, NC 27834, USA

## A Perspective on “Skeletal Muscle Nuclei in Mice are not Postmitotic”

As I think about my time in graduate school or as a postdoc, I remember reading countless papers with some version of the following phrase “skeletal muscle is postmitotic. . .” or “DNA synthesis does not occur after fusion. . .” Collectively these statements are something I always accepted as proven and something I would venture to say that most individuals, who study skeletal muscle would consider dogma. Thus, reading the work of Borowik et al.^1^ in this issue of *Function*, caused me to stop and really focus on the data because the ideas challenged these very same concepts. Perhaps, this illustrates the dangers of the word “dogma” in science. As previously suggested by others, it may be better to think that the concept of postmitotic myonuclei was never dogma but instead a paradigm of understanding based on a wealth of published evidence.^2^ The paradigm of postmitotic myonuclei was established across multiple labs using a variety of scientific approaches, which provided confidence to the field that paradigm was valid due to the high degree of rigor^3–6^.

The work in this issue of *Function* by Borowik et al.,^1^ demonstrates increases in DNA synthesis in myonuclei, which would suggest myonuclear replication is occurring. Within the manuscript, the authors provide a synopsis describing a sequence of publications that led them to test if DNA synthesis may be occurring in myonuclei. Specifically, the authors had published papers describing increases in DNA synthesis in skeletal muscle across a variety of models (ie, exercise in humans or mechanical stimulation of muscle in mice). Although not proven, the authors assumed that satellite cell expansion explained the DNA synthesis measures. Thus, in this current study, the authors used a genetic mouse model where satellite cells were ablated, and they hypothesized that no increases in DNA synthesis should be detected. Surprisingly, the data indicated an increase in DNA synthesis even when the satellite cells were ablated, which the authors interpreted to mean that the increase was due to proliferation of nonmuscle cells. Before proceeding to nonmuscle cells, the authors sought to rule out myonuclei as the source of DNA synthesis. To accomplish this, the authors developed a mouse model where a skeletal muscle-specific Tet-On mouse (HSA-rtTA) was crossed with a tetracycline-response element histone 2B-green fluorescent protein mouse (TRE-H2B-GFP). Using this mouse, allowed the investigators the ability to sort the GFP^+^ myonuclei and sort the GFP^−^ nuclei (from nonmuscle cells) into two distinct fractions. The authors confirmed the ability to separate two fractions using multiple different approaches. Upon confirmation that isolation of myonuclei was possible, they then delivered deuterium oxide (D_2_O) to the animals, which will only incorporate into DNA using de novo pathways ruling out any signal accumulation due to DNA repair. After the D_2_O exposure, the investigators were able to isolate the different fractions and directly measure D_2_O labeling. Under normal cage conditions, the authors found that 10% of the total DNA synthesis occurred in the GFP^+^ fraction (ie, myonuclei); however, if the authors induced muscle hypertrophy, a significant increase in DNA synthesis was detected in the GFP^+^ fraction suggesting that the myonuclei are replicating. Using mathematical predictions, their data suggest that in resting conditions 2.5%–8% of the resident nuclei replicate their DNA in a year, which on face value seems low; however, when the muscle was loaded the increase equated to hundreds to thousands of replication events over a 2.5-mo period. These results are quantitively similar to work on myonuclei response to denervation.^7^ Thus, the data suggest that myonuclei are not postmitotic. In the paper, the authors employed multiple different approaches coupled with a variety of validation techniques, which resulted in a rigorous study that is expected to induce a substantial amount of scientific discussion.

Challenging dogma is a hard road and often the road less traveled for a variety of reasons. Scientists as a community can be critical and/or skeptical and many will likely react this way to the work. These reactions are fine and should be expected, but the work should also provide thoughtful interactions and inspiration to others with the appropriate expertise or research tools to further assess these ideas. Can they confirm the potential for myonuclei proliferation in skeletal muscle using other approaches? Does this mean the data suggest that myonuclei replication or DNA synthesis in myonuclei occurs during all moments where satellite cells are thought to be necessary? For example, muscle regeneration, development, or postnatal muscle growth? It is important that the work is given context to the field. At this point, a lot remains unknown, and the field needs to consider next steps with respect to these results. Will this paper be an impetus for researchers to refine the previously established paradigm concerning myonuclei being postmitotic? The beauty of science is it allows for change and refinement as our methods and techniques advanced and/or improve. The ability to redefine paradigms is important to advance our understanding of physiological mechanisms and it should be expected that it will occur as our ability to measure biological phenomena improves.

For me personally, the data forced me to stop, think, and reconsider what I know about muscle. The reaction induced by the work is a key characteristic of papers I find enjoyable and interesting to read. I do not always have to agree with investigators interpretations, but if the results cause me to really think and reconsider my own work then for me it often becomes a memorable paper. The work of Borowik et al.^1^ definitely met that criterion for me.

## Funding

None declared.
